# Comparison of the Treatment Response of Drug-Eluting Bead Transarterial Chemoembolization and Conventional Transarterial Chemoembolization in Patients With Hepatocellular Carcinoma

**DOI:** 10.7759/cureus.41701

**Published:** 2023-07-11

**Authors:** Hatem Adel, Muhammad K Shazlee, Saqib Qamar, Syed Muhammad Shahnawaz Hyder, Abdul Razaque

**Affiliations:** 1 Radiology, Indus Hospital and Health Network, Karachi, PAK

**Keywords:** liver cancer, tace, response, transarterial chemoembolization, hepatocellular carcinoma

## Abstract

Introduction

Hepatocellular carcinoma (HCC) is a common primary hepatic cancer. Its early diagnosis can aid in its treatment by curative means such as surgery or ablation. Advanced-stage diagnosis limits these treatment options, and such cases can be treated with transarterial chemoembolization (TACE). Conventional transarterial chemoembolization (cTACE) and drug-eluting bead transarterial chemoembolization (DEB-TACE) are usually used, and follow-up response is evaluated using modified Response Evaluation Criteria in Solid Tumors (mRECIST) criteria. This study was done to compare the treatment response of cTACE and DEB-TACE in patients with HCC.

Materials and methods

A retrospective review of electronic medical records of all patients diagnosed with HCC from January 2021 to August 2022 who underwent cTACE or DEB-TACE was undertaken at the Department of Interventional Radiology, Indus Hospital and Health Network. Both male and female patients aged 18 years or above with Child-Pugh class A and B were included. DEB-TACE or cTACE was performed by a fellowship-trained interventional radiologist, and the response was evaluated at six weeks follow-up using mRECIST criteria.

Results

A total of 129 patients were included in this study, with a mean age of 54.1 ± 10.8 years. The mean size of HCC was 3.1 ± 1.7 cm. Seventy-eight (60.5%) patients underwent cTACE, and 51 (39.5%) underwent DEB-TACE. Out of the 78 patients who underwent cTACE, complete response (CR) was found in 28 (35.9%), partial response (PR) was found in 33 (42.3%), stable disease (SD) was found in 12 (15.4%), and progressive disease (PD) was found in five (6.4%) patients. Of the 51 patients who underwent DEB-TACE, CR was found in 13 (25.5%), PR was found in 20 (39.2%), SD was found in 11 (21.6%), and PD was found in seven (13.7%) patients.

Conclusion

The response rate of TACE in the form of complete or partial response was higher with a lower frequency of stable or progressive disease. cTACE has a high response rate as compared to DEB-TACE.

## Introduction

Among primary malignancies of the liver, hepatocellular carcinoma (HCC) is the most frequent and is also the third leading cause of cancer-related death [1]. Although there is improvement in imaging techniques to diagnose HCC at an early stage, many HCCs are still diagnosed at an advanced stage [2]. Early-diagnosed HCC can be treated with potentially curative means such as resection, ablation (via radiofrequency or microwave), or liver transplant [2]. Advanced-stage diagnosis limits the treatment by curative means such as surgery [3]; therefore, in such cases, transarterial chemoembolization (TACE) is opted for management.

TACE is a treatment option in which antitumor medication is injected together with embolization particles within the arteries supplying the tumor. This has a strong antineoplastic effect on the tumor and results in ischemic necrosis [4]. In conventional TACE (cTACE), the antitumor medication is mixed with lipiodol, and embolization is carried out using polyvinyl alcohol (PVA) particles and gelatin sponge (gelfoam), thus offering a favorable prognosis to HCC patients [5]. Drug-eluting bead TACE (DEB-TACE) is a newer methodology in which microspheres loaded with antitumor medication are injected into the lesion. This technique results in the sustained release of antitumor drugs maximizing ischemic necrosis [6].

In literature, mixed results related to the comparison of cTACE and DEB-TACE have been published. Some authors advocate that DEB-TACE is safe and effective in HCC patients and in some cases superior to cTACE [1]. However, others have reported no additional benefit of DEB-TACE; however, post-procedure pain and fever were higher in DEB-TACE patients [7]. To the best of our knowledge, no study has been conducted from a developing country comparing treatment response to cTACE and DEB-TACE. Therefore, this study was done to compare the treatment response of cTACE and DEB-TACE in patients with HCC and provides a developing country perspective on TACE for HCC.

## Materials and methods

A retrospective review of electronic medical records of all patients diagnosed with HCC from January 2021 to August 2022 who underwent cTACE or DEB-TACE was undertaken at the Department of Interventional Radiology, Indus Hospital and Health Network. The requirement for institutional approval was waived as a retrospective review of the medical records of the patients was performed. Both male and female adult patients aged 18 years or above diagnosed with hepatocellular carcinoma on the background of chronic liver disease with Child-Pugh class A and B were included. Patients who were Child-Pugh class C, who were already diagnosed with acute or chronic renal failure or hepatic failure, or presenting for follow-up after ablative therapy of HCC were excluded. The baseline characteristics of the patients, such as age, gender, and duration of symptoms, were also recorded. The size of the tumor pre-TACE was also recorded. All TACE procedures were performed in the catheterization lab by a fellowship-trained interventional radiologist with more than three years of experience.

Conventional transarterial chemoembolization (cTACE) was performed after the puncture of the right femoral artery, followed by super-selective cannulation of tumor vessels originating from the hepatic artery. For embolization, 50 mg of doxorubicin mixed with lipiodol was injected within the tumor, followed by injection of gelfoam or polyvinyl alcohol (PVA) particles, and embolization was stopped on the appearance of sluggish flow.

DEB-TACE was performed after the puncture of the right femoral artery, followed by super-selective cannulation of tumor vessels originating from the hepatic artery. For embolization, hepaspheres were initially loaded with 50 mg of doxorubicin diluted in water for injection. The chemotherapeutic agent mixture and hepaspheres were stored at room temperature for 30 minutes and mixed after every 10 minutes. Non-ionic contrast was also mixed. It was injected within the tumor vessels under angiographic guidance using a catheter at a speed of 1 mL per minute until sluggish flow was achieved. The response was evaluated as per modified Response Evaluation Criteria in Solid Tumors (mRECIST) criteria after a six-week follow-up.

Tumor response was labeled at the first follow-up at six weeks after TACE on contrast-enhanced triphasic CT scan. The treatment response was defined as per modified Response Evaluation Criteria in Solid Tumors (mRECIST) [8]. Complete response (CR) was labeled when no arterial enhancement was appreciated in all the visualized HCCs. Partial response (PR) was labeled when a decrease of approximately 30% or more in the sum of viable (arterially enhancing) HCCs was observed. Progressive disease (PD) was labeled when an increase of approximately 20% or more in the diameter of viable (arterially enhancing) HCCs was observed. Stable disease (SD) was labeled when no interval change was seen since the previous examination, i.e., disease that did not show CR, PR, or PD.

All statistical analyses were carried out using Statistical Package for the Social Sciences (SPSS) version 22 (IBM SPSS Statistics, Armonk, NY, USA). Quantitative variables such as age, duration of symptoms, and size of HCC were mentioned as mean and standard deviation (SD). Qualitative variables such as gender and mRECIST response were mentioned as frequency and percentage. Effect modifiers such as age, gender, and symptom duration were controlled through stratification. Post-stratification chi-square test was applied, and a p value of less than or equal to 0.05 was taken as significant.

## Results

One hundred twenty-nine patients were included in this study, with a mean age of 54.1 ± 10.8 years. The mean duration of symptoms was 22.2 ± 7.7 weeks. Seventy-nine (61.2%) were males, and 50 (38.8%) were females. The mean size of HCC was 3.1 ± 1.7 cm. A total of 78 (60.5%) patients underwent cTACE, and 51 (39.5%) underwent DEB-TACE. Patient characteristics are summarized in Table 1.

**Table 1:** Baseline characteristics of the patients *Mean ± SD SD: standard deviation, HCC: hepatocellular carcinoma

	Number	%
Age, years	54.1 ± 10.8^*^	
Mean HCC size, cm	3.1 ± 1.7^*^	
Gender		
Males	79	61.2
Females	50	38.8
Type of transarterial chemoembolization		
Conventional	78	60.5
Drug-eluting beads	51	39.5
Child-Pugh classification		
A	12	23.5
B	32	62.7
C	7	13.7
Vascular invasion		
No	43	84.3
Yes	8	15.7
Extrahepatic spread		
No	48	94.1
Yes	3	5.9

Out of seventy-eight patients who underwent cTACE, complete response (CR) was found in 28 (35.9%), partial response was found in 33 (42.3%), stable disease was found in 12 (15.4%), and progressive disease was found in five (6.4%) patients (Table 2).

**Table 2:** Treatment response to conventional transarterial chemoembolization (n = 78)

	Number	%
Complete response	28	35.9
Partial response	33	42.3
Stable disease	12	15.4
Progressive disease	5	6.4

Among 51 patients with DEB-TACE, CR was found in 13 (25.5%), PR was found in 20 (39.2%), SD was found in 11 (21.6%), and PD was found in seven (13.7%) patients (Table 3).

**Table 3:** Treatment response to drug-eluting bead transarterial chemoembolization (n = 51)

	Number	%
Complete response	13	25.5
Partial response	20	39.2
Stable disease	11	21.6
Progressive disease	7	13.7

In patients who underwent cTACE, the response was higher in male patients (p value = 0.762), in patients aged more than 40 years (p value = 0.723), and in patients with an HCC size of >2 cm (p value = 0.560) (Table 4).

**Table 4:** Comparison of outcome by conventional transarterial chemoembolization with patient characteristics *Chi-square test applied HCC: hepatocellular carcinoma

	Response				Total	p value
	Complete response	Partial response	Stable disease	Progressive disease		
Gender						
Female	9 (11.5%)	13 (16.7%)	6 (7.7%)	2 (2.6%)	30 (38.5%)	0.762^*^
Male	19 (24.4%)	20 (25.6%)	6 (7.7%)	3 (3.8%)	48 (61.5%)	
Age						
≤40 years	5 (6.4%)	6 (7.7%)	1 (1.3%)	1 (1.3%)	13 (16.7%)	0.723^*^
>40 years	23 (29.5%)	27 (34.6%)	11 (14.1%)	4 (5.1%)	65 (83.3%)	
Duration of symptoms						
≤14 weeks	3 (3.8%)	6 (7.7%)	2 (2.6%)	0 (0%)	11 (14.1%)	0.658^*^
<14 weeks	25 (32.1%)	27 (34.6%)	10 (12.8%)	5 (6.4%)	67 (85.9%)	
Size of HCC						
≤2 cm	9 (11.5%)	13 (16.7%)	6 (7.7%)	3 (3.8%)	31 (39.7%)	0.560^*^
>2 cm	19 (24.4%)	20 (25.6%)	6 (7.7%)	2 (2.6%)	47 (60.3%)	
Vascular invasion						
No	24 (30.8%)	23 (29.5%)	12 (15.4%)	5 (6.4%)	64 (82.1%)	0.060^*^
Yes	4 (5.1%)	10 (12.8%)	0 (0%)	0 (0%)	14 (17.9%)	
Extrahepatic spread						
No	26 (33.3%)	28 (35.9%)	12 (15.4%)	4 (5.1%)	70 (89.7%)	0.385^*^
Yes	2 (2.6%)	5 (6.4%)	0 (0%)	1 (1.3%)	8 (10.3%)	

Similarly, in patients who underwent DEB-TACE, response was higher in male patients (p value = 0.492), in patients aged more than 40 years (p value = 0.714), and in patients with an HCC size of >2 cm (p value = 0.177) (Table 5).

**Table 5:** Comparison of outcome by drug-eluting bead transarterial chemoembolization with patient characteristics *Chi-square test applied HCC: hepatocellular carcinoma

	Response				Total	p value
	Complete response	Partial response	Stable disease	Progressive disease		
Gender						
Female	6 (13.3%)	6 (13.3%)	3 (6.7%)	0 (0%)	16 (35.6%)	0.492^*^
Male	7 (15.6%)	14 (31.1%)	7 (15.6%)	1 (2.2%)	29 (64.4%)	
Age						
≤40 years	1 (2.2%)	3 (6.7%)	0 (0%)	0 (0%)	4 (8.9%)	0.714^*^
>40 years	12 (26.7%)	17 (37.8%)	10 (22.2%)	1 (2.2%)	41 (91.1%)	
Duration of symptoms						
≤14 weeks	0 (0%)	9 (20%)	0 (0%)	0 (0%)	9 (20%)	0.007^*^
<14 weeks	13 (28.9%)	11 (24.4%)	10 (22.2%)	1 (2.2%)	36 (80%)	
Size of HCC						
≤2 cm	2 (4.4%)	10 (22.2%)	2 (4.4%)	0 (0%)	14 (31.1%)	0.177^*^
>2 cm	11 (24.4%)	10 (22.2%)	8 (17.8%)	1 (2.2%)	31 (68.9%)	
Vascular invasion						
No	11 (24.4%)	16 (35.6%)	9 (20%)	1 (2.2%)	37 (82.2%)	0.752^*^
Yes	2 (4.4%)	5 (11.1%)	1 (1.1%)	0 (0%)	8 (17.8%)	
Extrahepatic spread						
No	13 (28.9%)	19 (42.2%)	9 (20%)	1 (2.2%)	42 (93.3%)	0.693^*^
Yes	0 (0%)	2 (4.4%)	1 (2.2%)	0 (0%)	3 (6.7%)	

## Discussion

Modified Response Evaluation Criteria in Solid Tumors (mRECIST) is used largely by researchers to evaluate the endpoints of treatment for HCC. These treatments usually include ablative techniques such as radiofrequency ablation (RFA) or microwave ablation (MWA) or include treatment by chemoembolization or radioembolization such as TACE or transarterial radioembolization (TARE) [9]. When evaluating responses based on RECIST or mRECIST criteria, it is usually advisable to use the same imaging modality. Magnetic resonance imaging (MRI) of the abdomen with contrast is the standard imaging modality for pretreatment and posttreatment monitoring of disease. Moreover, for HCC, PET/CT is not included in the treatment response evaluation [10-12].

Various transarterial treatment modalities exist for treating HCC. Selective internal radiation therapy (SIRT), transarterial radioembolization (TARE), and transarterial chemoembolization (TACE) are included in these treatment methods. TACE is the most widely used method. In SIRT, yttrium-90 microspheres are infused intra-arterially for radioembolization [13,14].

In oncology, overall survival (OS) is usually the primary endpoint to evaluate cancer treatment results. However, due to the variable amount of treatments available for HCC, alternative endpoints such as progression-free survival (PFS), time to progression (TTP), and objective response rate (ORR) are surrogate endpoints adopted for assessing HCC response to locoregional treatments [15,16]. ORR is usually labeled as combining the results of CR and PR. Moreover, ORR usually evaluates the intervention benefit before the administration of additional medications for HCC. PFS almost also does similar.

Our study results have shown a high rate of complete and partial response in patients with HCC treated with TACE. This contrasts with the one reported in an international study in which the complete and partial response rate was lower [17]. This difference could be attributed to a difference in sample size; in our study, the sample size was larger. Moreover, the difference could also be related to inclusion criteria [17]. It could be also hypothesized that this difference could be due to the difference in using response evaluation criteria [17]. In our study, mRECIST criteria were used, whereas the previous study utilized the European Association for the Study of the Liver (EASL) criteria [17].

Our study results have further shown that cTACE is more effective and has a high ORR as compared to DEB-TACE. This contrasts with the results reported in an international study that reports the superiority of DEB-TACE over cTACE [18]. This difference could be hypothesized as a result of a slightly larger sample size of the international study [18]. Moreover, in that study, the proportion of patients undergoing DEB-TACE was higher as compared to cTACE; however, in our study, the proportion of cTACE patients was higher.

A regional study was conducted to compare the treatment response of DEB-TACE and cTACE [19]. The study was conducted on infiltrative HCCs. The results of that study show that in patients with follow-up at one month, DEB-TACE achieved higher disease control as compared to cTACE. However, our study shows that cTACE had a high ORR. This result could be attributed to the difference in follow-up duration. Another reason could be due to the difference in the study population selected. Moreover, that study showed no difference in results at three-month follow-up duration [19].

In the early and very early stages of HCC, ablative methods predominate as treatment modalities. However, an international study compared the use of DEB-TACE and cTACE in such cases [20]. That study was conducted on a small sample size of 40 in comparison to a larger sample size of 129 in our study. Moreover, the study results show superior results of DEB-TACE in HCC response in comparison to cTACE. A difference in sample size could be postulated as a reason for the difference in results. Moreover, the difference in study population ethnicity could also be a possible reason.

Another meta-analysis compared DEB-TACE with cTACE. Their results showed that the therapeutic effects of DEB-TACE and cTACE are similar [21]. Another meta-analysis showed that the mean peak plasma concentration of doxorubicin is higher in DEB-TACE as compared to cTACE, thereby making DEB-TACE more effective [22].

Our study results are not without certain limitations. We did not include infiltrative HCCs in our study. Studies have shown a good treatment response of HCC to TACE. Moreover, PFS was not studied in our study. PFS is a newly emerging oncological endpoint for HCC evaluation. Another newly emerging oncological endpoint for HCC is TTP, which was also not analyzed in our study and therefore counts as a limitation. Also, complications as a result of TACE were not studied. It is reported that DEB-TACE is better tolerated than cTACE [19]. Certain confounding factors such as the Eastern Cooperative Oncology Group (ECOG) status, serum alpha-fetoprotein (AFP) levels, and the cause of chronic liver disease were not evaluated. An additional limitation of our study was that we did not include the status of immunotherapy. We believe that these variables may affect treatment outcomes for HCC. Therefore, it is recommended that further studies should be carried out incorporating these variables to further obtain insight related to the success of these treatments in our population.

## Conclusions

This study evaluates and compares the response of conventional and drug-eluting bead transarterial chemoembolization in the management of hepatocellular carcinoma. To the best of our knowledge, being the first of its kind study from a developing country, it shows a good response rate of HCC to treatment by cTACE. Therefore, our results highlight that the value of cTACE should not be ignored in the management of HCC. In patients with advanced disease, TACE can provide a favorable treatment option and can aid in the improvement of disease prognosis.
